# Cognitive, Parent and Teacher Rating Measures of Executive Functioning: Shared and Unique Influences on School Achievement

**DOI:** 10.3389/fpsyg.2017.00048

**Published:** 2017-01-30

**Authors:** Marielle C. Dekker, Tim B. Ziermans, Andrea M. Spruijt, Hanna Swaab

**Affiliations:** ^1^Department of Clinical Child and Adolescent Studies, Faculty of Social and Behavioural Sciences, Leiden UniversityLeiden, Netherlands; ^2^Leiden Institute for Brain and Cognition, Leiden UniversityLeiden, Netherlands

**Keywords:** working memory, inhibition, shift, math, spelling

## Abstract

Very little is known about the relative influence of cognitive performance-based executive functioning (EF) measures and behavioral EF ratings in explaining differences in children's school achievement. This study examined the shared and unique influence of these different EF measures on math and spelling outcome for a sample of 84 first and second graders. Parents and teachers completed the Behavior Rating Inventory of Executive Function (BRIEF), and children were tested with computer-based performance tests from the Amsterdam Neuropsychological Tasks (ANT). Mixed-model hierarchical regression analyses, including intelligence level and age, showed that cognitive performance and teacher's ratings of working memory and shifting concurrently explained differences in spelling. However, teacher's behavioral EF ratings did not explain any additional variance in math outcome above cognitive EF performance. Parent's behavioral EF ratings did not add any unique information for either outcome measure. This study provides support for the ecological validity of performance- and teacher rating-based EF measures, and shows that both measures could have a complementary role in identifying EF processes underlying spelling achievement problems. The early identification of strengths and weaknesses of a child's working memory and shifting capabilities, might help teachers to broaden their range of remedial intervention options to optimize school achievement.

## Introduction

Executive functions (EFs) are generally defined as effortful cognitive abilities that help plan, guide and control goal-directed mental processes and behavior. Executive control is assumed to be involved in both math and spelling performance. Math calls for executive control to select and manipulate relevant numbers, to disregard irrelevant information, to choose the right computational methods, to temporarily store and manipulate numbers and other information, and to be able to switch between various procedures or operations (e.g., Raghubar et al., 2010; Friso-van den Bos et al., 2013; Yeniad et al., 2013; Cragg and Gilmore, 2014). Written spelling requires understanding in the language forms (i.e., morphology), sound structures, word meanings, and origins. Written spelling is also assumed to require executive control in order to efficiently integrate phonological, orthographical, and morphological information, and motor planning (Berninger et al., 2006; Garcia et al., 2010; Preßler et al., 2013).

The observation that EF abilities mature at different rates over time and have their peaks at different ages, suggests that EF incorporates separable abilities (e.g., Klenberg, 2001; Davidson et al., 2006; Simonds et al., 2007; Best et al., 2009; Best and Miller, 2010). In many studies of school-aged children, there is an agreement that there are at least three fundamental EF abilities that are interrelated, but distinguishable: working memory, inhibitory control, and cognitive shifting or cognitive flexibility (e.g., Miyake et al., 2000; Jacob and Parkinson, 2015).

Working memory (WM) refers to the ability to temporarily store, manipulate and control incoming information at the same time. WM improves gradually during childhood and adolescence in a linear fashion (Best et al., 2009; Best and Miller, 2010). Inhibitory control allows for the suppression of actions and resistance to interference from irrelevant stimuli entering the WM and is considered to be a precondition for other EFs. During the preschool years, inhibition skills improve rapidly and around age four children show basic inhibitory control. These skills gradually and linearly improve between ages five to eight and further refinements in accuracy and speed occur in middle childhood and in adolescence (Best et al., 2009; Best and Miller, 2010; Clark et al., 2010). Shifting or cognitive flexibility refers to the ability to flexibly switch between strategies, rules, tasks or mental states. Both WM and inhibition skills are needed to shift effectively and efficiently (Garon et al., 2008; Best and Miller, 2010). Shifting ability develops from preschool years through adolescence (Best et al., 2009; Best and Miller, 2010).

Most research on the influence of EF on school achievement focuses on performance-based measures of EF (e.g., Allan et al., 2014). Cognitive performance-based EF tasks tend to measure the efficiency of information processing mechanisms of the brain. WM capacities in children have been clearly linked to math skills (e.g., DeStefano and LeFevre, 2004; Raghubar et al., 2010; Friso-van den Bos et al., 2013; Gerst et al., 2015). In two meta-analyses, inhibitory control has also been positively linked to various math skills in preschoolers and kindergartners (Allan et al., 2014) and in primary school-aged children (Friso-van den Bos et al., 2013), and also in recent studies a significant association between inhibition and math performance has been found (e.g., Gerst et al., 2015; Ten Eycke and Dewey, 2016). In two meta-analyses, shifting was associated with math skills in primary school-aged children (Friso-van den Bos et al., 2013; Yeniad et al., 2013). A recent study by Gerst et al. (2015) also reported a significant and positive relation between math and shifting.

A varying amount of research has been performed on the relation between cognitive measures of EF and spelling outcome, with most studies on WM, and only a few on inhibition or shifting. Studies on WM in relation to spelling skills show a positive association (e.g., Jongejan et al., 2007; Malstädt et al., 2012; Cardoso et al., 2013; Fischbach et al., 2013; Preßler et al., 2013; Becker et al., 2014; Re et al., 2014; Bexkens et al., 2015). Both inhibition (Altemeier et al., 2008) and shifting (Altemeier et al., 2008) have also been positively linked to spelling in first to fourth graders. Although cognitive EF performance is associated to cognitive performance in math and spelling, it remains unclear whether cognitive measures of EF are the best option to explain the more complicated, more demanding, and less structured performance situations at school where factors like fear and motivation also play an important role. Cognitive EF measures tend to neglect the effects of motivation, goals, and beliefs on EF, and their use in predicting quality of cognitive learning is complicated by task impurity problems (Salthouse et al., 2003). EF functioning is thought to be visible in everyday life whenever planning, problem solving, inhibition or troubleshooting is challenged. One might ask whether daily executive functioning at school or at home is also related to math and spelling performance. This would indicate the pervasive influence of EF on school performance on several levels of control.

Behavioral ratings of EF were developed to assess the application of EF skills in typical performance situations at home or at school and are assumed to be more ecologically valid. However, studies relating behavioral measures of EF to school achievement are limited. A significant association between behavioral WM problems and poorer math outcome has been reported by some (Clark et al., 2010; Gerst et al., 2015), but not by others (Ten Eycke and Dewey, 2016). Behavioral inhibitory problems have been found to show either a significant association (Clark et al., 2010; Gerst et al., 2015) or no association with math (Ten Eycke and Dewey, 2016). Behavioral problems with shifting have also been related to poorer math outcome (Gerst et al., 2015; Ten Eycke and Dewey, 2016). To our knowledge, only one study reported on the association between spelling and behavioral EF (teacher report) and showed that behavioral aspects of memory, shifting, and inhibitory control were related to children's spelling outcome in kindergarten and first grade (Kent et al., 2014). Nevertheless, behavioral ratings are challenged by rater bias (e.g., the halo effect, central tendency bias, leniency bias) and situational specificity of behavior, resulting in low cross-informant agreement (Achenbach et al., 1987). Furthermore, the high correlations between the different subscales also point to scale-impurity problems, questioning whether general behavioral impairment is being measured rather than different aspects of executive dysfunctioning (McAuley et al., 2010).

Both cognitive performance-based EF measures and behavioral EF rating measures clearly have their pros and cons. Results from a recent review study on the association between these EF measures in 13 studies using the Behavior Rating Inventory of Executive Function (BRIEF; Gioia et al., 2000), showed that only 19% of the reported correlations were significant with a median correlation of 0.18 (Toplak et al., 2013). It is evident that measures assessing cognitive and behavioral EF across informants tap into different aspects of EF. Meta-analytical evidence on inhibitory control in preschoolers and kindergartners (Allan et al., 2014), showed that the mean association between math achievement and inhibition was stronger for performance tasks (*r* = 0.35) compared to other-reports (*r* = 0.22). However, it is not yet clear how these different EF measures concurrently relate to real world external criteria like school achievement. Understanding to what extent different EF measures share variance and add unique variance in relation to school achievement could verify their validity and could provide us with a more balanced view of relevant EF aspects.

Thus far, only the studies of Gerst et al. (2015) and Miranda et al. (2015) provide some insight into the relative impact of these different types of EF measures on school outcome, although math outcome was only studied by Gerst et al. (2015) and neither of these two studies looked at spelling. Gerst et al. (2015) examined both cognitive EF measures and teacher behavioral EF rating measures of WM, inhibition and shifting and found moderate correlations for all measures with math and reading comprehension outcome. Analyzing the shared and unique influence of these cognitive and behavioral measures for each EF in a full model with relevant covariates showed that both types of WM measures were complementary in the prediction of math and reading comprehension outcome. However, for inhibition and shifting, the behavioral EF rating did not add any unique variance to the prediction of math by the performance measure. In contrast, for reading comprehension, the cognitive measures for inhibition and shifting did not add any unique variance to the teacher rating. Miranda et al. (2015) concluded that teacher's global EF rating was more strongly related to reading accuracy and speed then parent's global EF rating.

A key issue when examining the impact of EF on school achievement is to what extent it is independent from intelligence (IQ). There is some evidence that IQ has associations with WM (Mahone et al., 2002; Friedman et al., 2006; Alloway and Alloway, 2010), inhibition (Mahone et al., 2002) and shifting (Ardila et al., 2000; van der Sluis et al., 2007), and that this relationship is partially attributable to shared executive or non-executive processing demands (e.g., processing speed) underlying both EF and IQ assessment (van der Sluis et al., 2007), as well as to shared method variance reflected in the ability to take tests in the case of performance based EF tasks. Some studies did indeed show that EF shared a lot of variance with IQ in predicting school achievement (e.g., Bull and Scerif, 2001; Espy, 2004). However, other studies, have shown that both performance-based and rating-based EF measures were uniquely related to school achievement after taking into account the possible confounding effects of intelligence (e.g., George and Greenfield, 2005; Alloway and Alloway, 2010; Preßler et al., 2013; Yeniad et al., 2013; Gerst et al., 2015; Dekker et al., 2016). These latter findings suggest that traditional intelligence tests might not assess abilities that are considered important from a neurocognitive perspective, and that IQ cannot be considered a proxy of EF or vice versa. However, the mixed findings point to the need to study the possible confounding effect of intelligence level.

The aim of this study was to examine the shared and unique influence of three different types of EF measures, i.e., performance-based, teacher's rating-based, and parent's rating-based, on math and spelling outcome in first and second graders, while taking level of intelligence into account. Based on the presented evidence we expected cognitive measures of WM, inhibition and shifting to be related to math and spelling. Because there are only a couple of studies, with contradicting results, concerning behavioral EF measures as markers for math and spelling differences, our expectations were tentative. Nevertheless, we assumed that behavioral executive dysfunctioning had a negative association with math and spelling outcome. Based on the findings of Gerst et al. (2015), we expected cognitive measures of EF to have the biggest impact on math outcome, except for WM where we predicted the behavioral rating-based measure would add unique variance. Based on Gerst et al. (2015) findings on reading comprehension, we tentatively assumed that behavioral EF ratings would have the biggest impact on our language related spelling outcome, except for WM for which the cognitive measure was also expected to add unique variance. We further assumed that teacher's ratings of EF would have a bigger association with school achievement than parent's EF ratings (Miranda et al., 2015), as EF demands at home are different then EF demands at school, with the latter being more likely to be related to school readiness, attitude toward learning and testing, and thus with school achievement.

## Methods

### Procedure

The current study is part of an ongoing pretest-posttest intervention study called “Curious Minds' that focuses on neurocognitive, social, and environmental factors affecting children's” learning at school and at home. Children were recruited from two primary schools in the Dutch province of Zuid-Holland during November 2013 (school 1) and March 2014 (school 2). The Ethical Board of the department of Education and Child studies at Leiden University has given ethical approval for this study (ECPW-2010016).

Only children in grade 1 or 2, all aged 6–8 years, were included in this study. All parents of students from grade 1 or 2 (*N* = 172) received written information about the study from their child's school and were invited to attend an informational meeting. Written informed consent was obtained from all 105 parents who participated (response = 61.0%). Chi-square tests with a continuity correction showed no significant differences between participants and non-participants in gender, grade, or school (all *p* > 0.05), neither did a *t*-test for age (*p* > 0.05).

All parents and teachers were asked to complete a questionnaire on their child's or student's behavioral EF. Cognitive EF data was collected during school visits. Each child completed several computer-based performance-based EF tasks. Each assessment period lasted about an hour and a half and took place in a quiet room to minimize distraction. All assessments were done by the researchers or by Master's students who completed an extensive training in test administration, including video-feedback sessions. Pretest data was collected in the period between November 2013 and February 2014 (school 1), and May and June 2014 (school 2). Intelligence level was assessed during the post-test data collection phase. As IQ is considered to be quite stable over time, we expected that the time between this study's pre- and post-test of about half a year, would be of negligible influence (Canivez and Watkins, 1998). Dutch standardized paper-and-pencil achievement tests scores used to monitor math and spelling progress were retrieved from each school's records at pretest. We obtained full achievement test score information, full cognitive EF data and teacher EF ratings for 104 out of the 105 participating children, for 103 children we were able to estimate intelligence level, and we received 86 EF ratings from parents. Complete data for this study was available for 84 children (80.0% of all participating children; 48.8% of all eligible children) from 7 different classes. Children with complete data did not significantly differ from children without complete data (*N* = 21) on age, grade, school or gender (all *p* > 0.05).

### Measures

#### Cognitive EF

Cognitive EF was measured with three neuropsychological tasks from the Amsterdam Neuropsychological Tasks (ANT, version 2.0; De Sonneville, 1999, 2011). The ANT has been used extensively to examine EF and related cognitive processes in various clinical and non-clinical populations and has high sensitivity for neuropsychological problems as well as good reliability and appropriate validity (De Sonneville, 2005, 2014; Rowbotham et al., 2009). All computer tasks were preceded by instructions from the test leader and practice trials. All test stimuli were presented on a computer screen and the child had to respond by pressing a mouse key.

##### Working memory

Visuospatial working memory was measured with the ANT Spatial Temporal Span (STS–part 2)—backward span. In this task, nine squares are presented on the computer screen in a three-by-three matrix. During each trial, an incremental sequence of these squares (two up to a maximum of nine) is pointed out by a hand animation. Each sequence of appointed squares is presented in two successive trials. The participant is instructed to repeat this sequence by clicking the same squares in reverse order. In each trial the sequence is preceded by an auditory cue (a beep). The task aborts automatically whenever two successive trials of the same sequence number are incorrect. The number of correct identified targets in correct order backwards was used as a measure of visuospatial working memory.

##### Inhibition

Inhibition of a prepotent ongoing motor response was assessed with the ANT Go-NoGo (GNG–biased) task. In the GNG task the mouse button has to be clicked whenever a yellow square with a hole at the bottom is displayed (the Go signal; 75% of the trials). Whenever a full yellow square is displayed (the NoGo signal; 25% of the trials) the child has to withhold the prepotent motor response and do nothing. The number of false alarms on the 18 NoGo trials was used as a measure of level of inhibition. A higher amount of false alarms (e.g., the participant clicks when the target signal is not presented) indicates that a child is less able to stop an ongoing response.

##### Shifting

Shifting was assessed with the ANT Response Organization Objects (ROO–part 3)—mixed compatible and incompatible. During the third part of the ROO task, the color of the ball alternates randomly between green and red and the child has to shift between response sets. Whenever the green ball appears a compatible dominant response is required (click the mouse button that corresponds to the side where the green ball is presented) and when the red ball appears an incompatible subdominant response is required (click the mouse button on the opposite side of where a red ball is presented). This part consists of 80 trials; 40 trials requiring a compatible response and 40 trials requiring an incompatible response. The overall amount of errors in part 3 was used to measure level of visuospatial shifting.

#### Behavioral EF

Behavioral EF was measured with BRIEF (Gioia et al., 2000; Huizinga and Smidts, 2009, 2010). Both the teacher's form (BRIEF-teacher) and the parent's form (BRIEF-parent) were used. The BRIEF teacher's form assesses everyday behavioral EF problems in the classroom and the BRIEF parent's version does the same for the home situation. Fifteen different classroom teachers filled out 5–9 BRIEF-teacher questionnaires (mean = 5.6; mode = 4; *SD* = 1.6). The BRIEF has satisfactory internal consistency, test-retest reliability, moderate inter-rater agreement and appropriate evidence of predictive and discriminant validity and is used for children from 5 to 18 years old. The BRIEF contains 86 items that make up eight scales that form a Behavior Regulation Index. In this study we used the raw scale score of the Working Memory, the Inhibit, and the Shift scale. A higher BRIEF scale score indicates a higher level of executive dysfunction.

##### Problems with working memory

The Working Memory scale (WM) of the BRIEF assesses the capability to hold information when completing a task, when encoding information, or when generating goals/plans in a sequential manner (e.g., forgets what he/she was doing, trouble remembering things, losing track of what they are doing).

##### Problems with inhibitory control

The Inhibit scale of the BRIEF assesses the amount of trouble a child has controlling impulses and to stop engaging in a behavior (e.g., gets out of control more than friends, has difficulty staying seated in the classroom, often interrupts others in class, requires more adult supervision).

##### Problems with shifting

The Shift scale of the BRIEF assesses the problems a child has with moving freely from one activity or situation to another, alternating attention or changing strategies (e.g., difficulty to flexibly solve problems, to make transitions, tolerate change, or shift attention).

#### Intelligence level

Level of intelligence (IQ) was estimated using the Vocabulary (V) and Block Design (BD) subtest of the Dutch Wechsler Intelligence Scale for Children 6–17 years old (WISC-III-NL) at post-test, about half year later (Kort et al., 2005). The short form estimates of full scale IQ for the WISC-III (FSIQ) were obtained according to the algorithm: 2.9 × (sum of normed scores) + 42; an algorithm based on Tellegen and Briggs's linear scaling technique (Tellegen and Briggs, 1967; Campbell, 1998). The WISC-III V-BD estimate has been found valid for the estimation of full scale IQ, given a sufficient corrected FSIQ validity (*r* = 0.82) and split-half reliability (*r* = 0.91) (Campbell, 1998). The 2.8 year stability of the WISC-III Vocabulary subtest has been found to be 0.75, and that of Block Design subtest 0.78 (Canivez and Watkins, 1998).

#### School achievement

To assess math and spelling ability we used the Dutch standard CITO Mathematics Test (CMT; Janssen et al., 2010) and CITO Spelling Test (CST; de Wijs et al., 2010). The CMT and the CST are both composite national curriculum paper-and-pencil achievement tests that are standardized and norm-referenced. They have good psychometric properties and are commonly used in Dutch schools to monitor the progress of students in primary education (de Wijs et al., 2010; Jansen et al., 2013). There are two different tests for each grade, one regularly administered halfway through the year (January) and one around June. We collected the CMT and CST scores through the schools at the time of the pretest. Therefore, in this study we used the January 2014 CITO tests scores from school 1, and the June 2014 CITO tests score from school 2. To allow for comparison between the students' math and spelling scores we used the age equivalent math score (AES) and subtracted the number of months of education the student had received up to that point (10 months per year, starting from grade 1). A positive score of 5 means that a student is about 5 months ahead in mathematical or spelling skills relative to the amount of education received up to that point in time (the general population AES mean is 0 months).

##### Mathematical abilities

The Dutch standard CITO Mathematics test (CMT) was used to assess various mathematical abilities (Janssen et al., 2010). In the current study's grades the following math skills are covered: (a) number and number relations; (b) addition and subtraction; (c) multiplication and division; and (d) measuring (e.g., weights, length, surface, time).

##### Spelling abilities

The Dutch standard CITO Spelling test (CST) was used to assess implicit spelling abilities (de Wijs et al., 2010). Spelling ability for the current study's age group is tested by having children write 50 words (January Grade 1) or sentences (June grade 1) dictated by their teacher. Starting from grade 2 there are two parts: (1) 25 dictated sentences; and (2). 25 questions where children have to pick out the sentence with the wrongly spelled word (in bold case) out of four different sentences. All CST scores are rescaled to make the CST comparable across children.

### Statistical analysis

Data was analyzed using simple correlations and with linear mixed-effects modeling using IBM SPSS version 23. All variables that were significantly skewed (SE > 3.0) were first log transformed (BRIEF Inhibit and Shift scale for both parent and teacher rating) or square root transformed (GNG number of false alarms, ROO number of errors part 3, BRIEF WM scale for both parent and teacher rating). A hierarchical mixed-model regression analysis, based on our hypotheses, with maximum likelihood estimation was used to test each hypothesized model explaining math or spelling achievement outcome. Analysis were performed for each type of EF (WM, inhibition, shifting) using all three methods (cognitive, teacher rating, and parent rating), and including IQ. A random intercept for class (*n* = 7) was included to control for the slight non independence of our data due to students being nested in classes (multi-level data). The intra class correlation (ICC = Variance (intercept)/(Variance(intercept) + Variance(error)) for the null model (intercept-only model) of math was 0.03 (3% of the variance was attributed to class level) and for spelling the ICC was 0.08. The difference in −2Log Likelihood, which follows a χ^2^ distribution with the difference in degrees of freedom between the two nested models as its degrees of freedom, between two adjacent nested models was calculated and also the Schwarz's Bayesian Information Criterion (BIC) difference. A BIC difference between two nested models can be considered a weak (0–2), a positive (2–6) or a strong (>6) indication for a better model (Raftery, 1995). A model was considered an improved model whenever the −2LL difference was significant (*p* < 0.05) and the BIC difference was bigger than 0. In each hierarchical model, IQ was entered first (model 1). For math outcome, the next model included the cognitive EF measure (Model 2). If this model was a significant improvement over the IQ only model, a model adding the corresponding teachers' EF rating was estimated (Model 3). The matching parent's EF rating was entered after the teacher's rating (Model 4). For spelling outcome, Model 2 included the teacher's EF rating. If this model was a significant improvement over the IQ only model, a model adding the corresponding cognitive EF measure was estimated (Model 3). The matching parent's EF rating was entered after the cognitive EF measure (Model 4). Whenever an EF measure would not significantly improve a previous model, we would replace this measure with the next EF counterpart measure (adding b or c to the model name). As only a small pool of not substantially correlated independent variables (see **Table 2**) were included in this study, we also ran a mixed-model stepwise backwards regression analyses. As similar results were found when using this method of model selection, we only report the hierarchical approach estimates in this paper, including fixed effect (intercept, regression weights) and the random effect estimates (variance around the intercept and random error). Effect sizes were interpreted as: I. a small ‘practically’ significant effect (r or β ≥ 0.2 and <0.5); II. a moderate effect (r or β ≥ 0.5 and <0.8) or III. a strong effect (r or β ≥ 0.8) (Ferguson, 2009).

## Results

### Sample description

Sample characteristics are shown in Table 1. Age (range 6–8 years) and gender (51.1% male) distributions were as expected. Children in this study were on average around 2 months ahead in math and spelling compared to a norm sample of Dutch peers, and had a somewhat higher estimated mean IQ score of 106. Comparing the educational level of the 164 parents in our sample to the educational level of the general Dutch population of 25- to 45-year-olds (*N* = 4,267,000), showed that the parents in our study were less likely to have a low educational level (11.6 vs. 33.6%; *z* = −5.96, *p* < 0.001), were more likely to have a medium educational level (48.8 vs. 28.3%; *z* = 5.83; *p* < 0.001), and equally likely to have a high educational level (39.6 vs. 38.1%; *z* = 0.40; *p* = 0.689) (CBS, 2013). Around 12% of the children were referred to mental health care in the past year (95% Confidence Interval = 5.0–18.8%) for the assessment and/or treatment of various developmental, emotional and behavioral problems (e.g., problems with attention and hyperactivity, anxiety, conduct related problems, pervasive developmental problems). This percentage is significantly higher than the 5.9% referral rate found in a large (*N* = 1710) Dutch general population study of 6–18-year-olds (*z* = −2.23, *p* = 0.026) (Tick et al., 2008). Teachers in our sample scored their students significantly more often in the clinical range of WM problems (T-score ≥ 65 = 20.2%) compared to 7% of the BRIEF-teacher Dutch norm sample of 5- to 8-year-olds (*N* = 55) (Z-score = −2.138, *p* = 0.032). No significant difference with the Dutch norm sample on the percentage of reported students in the clinical range was found for inhibition and shifting. Parents in our sample reported a similar percentage of children in the clinical range on all three BRIEF-parent scales compared to the Dutch BRIEF-parent norm sample of 5- to 8-year-olds (*N* = 311; all *p* > 0.05).

**Table 1:** **Demographic characteristics and descriptive statistics of independent and dependent variables**.

	**% (*N* = 84)**	**Mean (SD)**	**Scale range sample**
**DEMOGRAPHIC VARIABLES**			
Age^†^		87.54 (7.16) months	75–102 months
First grade	56.0		
School 1	67.9		
Males	48.9		
**Educational level parents**^‡^			
High	39.6		
Medium	48.8		
Low	11.6		
Mental Health Care referral past year	11.9		
**DEPENDENT VARIABLES**^¶^			
Math		2.67 (7.87) months	−22 to 26 months
Spelling		2.27 (6.45) months	−18 to 17 months
**INDEPENDENT VARIABLES**			
Full scale IQ estimate^§^		106.11 (12.17)	79.70–131.90
% < 85/% > 115	4.8/25.0		
**Cognitive EF measures: ANT (scale)**			
STS working memory (0–88)		31.45 (14.59)	4–75
GNG inhibit (0–18)		3.58 (2.49)	0–11
ROO-3 shift (0–80)		9.17 (9.48)	0–35
**Teacher behavioral rating scales: BRIEF-teacher (scale 10–30)**			
Raw score working memory		15.63 (5.12)	10–29
% T-score ≥ 65	20.2		
Raw score inhibit		13.50 (4.46)	10–30
% T-score ≥ 65	10.7		
Raw score shift		13.71 (3.70)	10–28
% T-score ≥ 65	17.9		
**Parent behavioral rating scales: BRIEF-PARENT (scale 10–30)**			
Raw score working memory		15.74 (4.44)	10–29
% T-score ≥ 65	4.8		
Raw score inhibit		15.73 (4.18)	10–30
% T-score ≥ 65	6.0		
Raw score shift		12.18 (3.21)	10–29
% T-score ≥ 65	6.0		

† *At time of Standardized CITO Math and Spelling test*.

‡ *% based on N = 164 parents using the Standard Classification of Education (SOI) 2006, edition 2014/15: “Low educational level (1),” including Primary and Lower secondary education (level 1 and 2 of the SOI); “Medium educational level (2),” including Upper secondary and Post-secondary non-tertiary education (level 3 and 4 of the SOI); “High educational level (3),” including Short cycle tertiary education and Bachelor's, Master's and Doctoral level (level 5–8 of the SOI; Dutch Central Bureau for Statistics [CBS], 2006, 2011)*.

¶ *Difference between achievement level (expressed as equivalent to number of months of education) and number of moths of education (10 months per grade)*.

§ *The short form (Vocabulary and Block Design) estimates of full scale IQ for the Wechsler Intelligence Scale For Children for children aged 6–8 years old (WISC-III-NL; Kort et al., 2005) were obtained according to the algorithm: 2.9 x (sum of normed scores) + 42 (Campbell, 1998). STS, Spatial Temporal Span (raw score number of identified targets in correct order backwards); GNG, raw score number of false alarms–biased; ROO-3, raw score number of errors compatible and incompatible part 3; BRIEF, Behavior Rating Inventory of Executive Function*.

### Correlations between EF, IQ, and school achievement

Correlations between all measures are reported in Table 2. Both standardized measures of math and spelling were significantly correlated with all three types of WM measures (|r| range = 0.28–0.43), which were significantly interrelated amongst themselves as well (|r| range = 0.25–0.31). Math and spelling were also significantly associated with the cognitive shifting measure, as was spelling with the teacher shifting problems rating. All effects were within the small range. None of the inhibition measures were related to school achievement. Parent-teacher cross-informant agreement of similar EFs were all significant and within the small range, while the cross-informant correlations between different types of EF were higher and in the moderate range. Intelligence level was significantly associated with math achievement (*r* = 0.41) and with the teacher's rating of WM problems (*r* = −0.31), but not with spelling achievement or any of the other EF measures. Furthermore, no significant correlation between age with any of the EF variables was found in this sample of 6–8 year olds.

**Table 2:** **Correlations between IQ estimate, executive function measures and standardized test scores for math and spelling (***N*** = 84)**.

	**Cognitive EF Measures**			**Behavioral EF Scales**						**IQ**	**School Achievement**		**Age**
				**BRIEF-parent**			**BRIEF-teacher**						
**Cognitive EF Measures**	**WM**	**Inhibit I**	**Shift**	**WM**	**Inhibit**	**Shift**	**WM**	**Inhibit**	**Shift**		**Math**	**Spelling**	
WM (STS)		*0.09*	−0.25^*^	−0.25^*^	−0.06	−0.15	−0.25^*^	0.04	−0.10	0.20	0.43^**^	0.37^**^	0.18
Inhibit I (GNG)			*0.01*	0.03	**0.10**	−0.07	−0.05	**0.01**	−0.09	0.04	0.15	0.03	−0.08
Shift (ROO-3)				0.19	0.03	**0.10**	0.19	0.10	−**0.01**	−0.19	−0.27^*^	−0.24^*^	0.03
**Behavioral EF Scales: BRIEF-parent**													
WM					0.60^**^	0.42^**^	0.31^**^	0.30^**^	0.27^**^	−0.12	−0.28^**^	−0.25^*^	0.18
Inhibit						0.60^**^	0.14	0.41^**^	0.31^**^	0.05	−0.08	−0.14	0.19
Shift							0.06	0.19	0.31^**^	−0.01	−0.11	−0.08	0.19
**Behavioral EF Scales: BRIEF-teacher**													
WM								0.57^**^	0.64^**^	−0.31^**^	−0.23^*^	−0.37^*^	−0.09
Inhibit									0.57^**^	−0.06	0.04	−0.09	−0.04
Shift										−0.07	−0.13	−0.24^*^	−0.06
IQ											0.41^**^	0.17	−0.18
Math												0.34^**^	−0.07
Spelling													−0.06

* p < 0.05;

** *p < 0.001. WM, working memory; BRIEF, Behavior Rating Inventory of Executive Function; STS, Spatial Temporal Span (number of identified targets in correct order backwards); GNG, Go-NoGo; ROO-3, Response Organisation Objects-part 3; Bold, monotrait–heteromethod correlations; Italic, heterotrait–monomethod correlations; regular, heterotrait–heteromethod correlations*.

### Math achievement: shared and unique influence of EF measures

In the best mixed models explaining math achievement (see Table 3), standardized math achievement was uniquely associated with intelligence level (b^*^ ranging from 0.34 to 0.38), the cognitive measure of WM (b^*^ (number correct) = 0.35), and the cognitive measure of shifting (b^*^ (number of errors) = −0.22), all with an effect size within the small range (see Table 3). None of the inhibition measures had a direct impact on math achievement. None of the teacher's or the parent's EF ratings added any unique variance to their cognitive EF counterpart in relation to math achievement. As age was uncorrelated with any of the outcome or the EF measures (see Table 2), including age in the analysis did not make a difference to the final results. Similar results for EF on math were found when IQ was excluded from the analysis, showing somewhat higher standardized regression weights for WM (b^*^ = 0.43) and shifting (b^*^ = −0.29), as shared variance with IQ was not corrected for.

**Table 3:** **Mixed model hierarchical regression analyses results of best model explaining MATH outcome (***N*** = 84) for each type of EF using multiple methods, IQ, and with random intercept for class (***n*** = 7)**.

**Independent variables within nested models^†^**	**−2LL (df) BIC**	**Nested Δ-2LL Δ BIC**	***p* (Δ nested model)**	**MATH: final model estimates**						
				**Fixed effects**			**Random effects**			
				***b (SE)***	b^*^	***p***	***Var* intercept**	***p***	***Var* error**	***p***
0. Null model	583.51 (3)			2.66 (1.00)		0.037	2.03 (4.5)	0.617	59.15 (9.58)	<0.001
ICC = 0.03	596.80									
**WM**										
Intercept				−26.88 (6.29)		<0.001	1.76 (3.05)	0.564	41.59 (6.74)	<0.001
1. IQ	567.04 (4)	16.47	<0.001	0.22 (0.06)	0.34	<0.001				
	584.77	12.03								
2. STS	554.38 (5)	12.66	<0.001	0.19 (0.05)	0.35	<0.001				
	576.53	8.24								
3a. BRIEF-t	554.15 (6)	0.23	0.632							
	580.74									
3b. BRIEF-p	550.67 (6)	3.74	0.053							
	577.26	−0.73								
**INHIBITION**										
Intercept				−25.99 (6.76)		<0.001	3.23 (4.14)	0.435	47.62 (7.71)	<0.001
1. IQ	567.04 (4)	16.47	<0.001	0.27 (0.06)	0.42	<0.001				
	584.77	12.03								
2a. GNG	564.66 (5)	2.38	0.123							
	586.81	−2.04								
2b. BRIEF-t	566.60 (5)	0.44	0.507							
	588.75	−3.98								
2c. BRIEF-p	566.34 (5)	0.70	0.403							
	588.50	−3.73								
**SHIFTING**										
Intercept				−19.66 (7.07)		<0.001	4.90 (4.85)	0.312	43.85 (7.09)	<0.001
1. IQ	567.04 (4)	16.47	<0.001	0.24 (0.06)	0.38	<0.001				
	584.77	12.03								
2. ROO-3	561.88 (5)	5.16	0.023	−4.37 (1.86)	−0.22	0.021				
	584.03	0.74								
3a. BRIEF-t	560.75 (6)	1.13	0.288							
	587.33	−3.30								
3b. BRIEF-p	560.91 (6)	0.97	0.325							
	587.49	−3.46								

† Whenever difference −2LL between fuller model minus adjacent nested more parsimonious model (lower number) = significant and BIC difference > 0, fixed and random estimates of best model are reported. ICC, intra class correlation; Δ-2RLL, −2Log Likelihood difference between two adjacent nested models (Δ df = difference in degrees of freedom between two adjacent nested models) following χ2 distribution; Δ BIC, difference in Schwarz's Bayesian Criterion between two adjacent nested models; p (Δ nested model), significance level improvement of adjacent more parsimonious model; b, regression weight; SE, Standard Error; b^*^, standardized regression weight; Var(intercept), variance attributed to class; Var(error), random error; WM, Working Memory; STS, Spatial Temporal Span; GNG, Go-NoGo; ROO-3, Response Organization Objects -part 3; BRIEF-p, Behavior Rating Inventory of Executive Functioning–parent rating; BRIEF-t, BRIEF-teacher rating.

### Spelling achievement: shared and unique influence of EF measures

The best mixed models for spelling outcome (see Table 4), showed that both teacher rated WM problems (b^*^ = −0.34) and the cognitive WM measure (b^*^(number correct) = 0.29) uniquely explained differences in spelling achievement, while IQ did not. A similar result was found for shifting, with both teacher rated problems with shifting (b^*^ = −0.24) and the cognitive shifting measure (b^*^(number of errors) = −0.27) accounting for spelling differences. All effects sizes were within the small range. None of the inhibition measures were related to spelling achievement, neither were any of the parent EF ratings. As age was uncorrelated with any of the outcome or the EF measures (see Table 2), including age in the analysis did not make a difference to the final results. Excluding IQ from of the model resulted in similar findings for EF with regard to spelling achievement.

**Table 4:** **Mixed model hierarchical regression analyses results of best model explaining SPELLING outcome (***n*** = 84) for each type of EF using multiple methods, IQ, and with random intercept for class (***n*** = 7)**.

**Independent variables within nested models^†^**	**−2LL (df) BIC**	**Nested Δ-2LL Δ BIC**	***p* (Δ nested model)**	**SPELLING: final model estimates**						
				**Fixed effects**			**Random effects**			
				***b (SE)***	b^*^	***p***	***Var* intercept**	***p***	***Var* error**	***p***
0. Null Model	548.31 (3)			2.21 (0.96)		0.054	3.24 (3.37)	0.335	37.75 (6.07)	<0.001
ICC = 0.08	561.60									
**WM**										
Intercept				7.58 (8.00)		0.346	4.29 (3.57)	0.229	27.81 (4.48)	<0.001
IQ^‡^	544.23 (4)	4.08	0.043	0.03 (0.05)	0.06	0.527				
	561.96	−0.36								
2. BRIEF-t	532.53 (5)	11.7	<0.001	−3.28 (1.09)	−0.32	0.003				
	554.69	7.29								
3. STS	525.00 (6)	7.53	0.006	0.12 (0.04)	0.28	0.006				
	551.58	3.11								
4. BRIEF-p	522.89 (7)	2.11	0.146							
	553.91	−2.33								
**INHIBITION**										
Intercept										
1. IQ	544.23 (4)	4.08	0.043							
	561.96	−0.36								
2a. BRIEF-t	543.56 (5)	0.67	0.413							
	565.72	−3.76								
2b. GNG	544.23 (5)	0.00	1.00							
	566.39	−4.43								
2c. BRIEF-p	542.06 (5)	2.17	0.141							
	564.21	−2.25								
**SHIFTING**										
Intercept				11.64 (9.67)		0.232	4.20 (3.67)	0.252	31.37 (5.05)	<0.001
1. IQ^‡^	544.23 (4)	4.08	0.043	0.08 (0.05)	0.16	0.119				
	561.96	−0.36								
2. BRIEF-t	540.30 (5)	3.93	0.047	−12.31 (6.37)	−0.22	0.040				
	562.46	0.50								
3. ROO-3	534.49 (6)	5.81	0.016	−3.89 (1.57)	−0.24	0.016				
	561.08	0.88								
4. BRIEF-p	534.45 (7)	0.04	0.841							
	564.46	−3.38								

† *Whenever difference −2LL between fuller model minus adjacent nested more parsimonious model (= lower number) = significant and BIC difference > 0, fixed and random estimates of best model are reported*.

‡ IQ is left in model to control for confounding even though BIC < 0. ICC = intra class correlation; Δ−2RLL = −2Log Likelihood difference between two adjacent nested models (Δ df = difference in degrees of freedom between two adjacent nested models) following χ^2^ distribution; Δ BIC, difference in Schwarz's Bayesian Criterion between two adjacent nested models; p (Δ nested model), significance level improvement of adjacent more parsimonious model; b, regression weight; SE, Standard Error; b^*^, standardized regression weight; Var(intercept), variance attributed to class; Var(error), random error; WM, Working Memory; STS, Spatial Temporal Span; GNG, Go-NoGo; ROO-3, Response Organization Objects-part 3; BRIEF-p, Behavior Rating Inventory of Executive Functioning–parent rating; BRIEF-t, BRIEF–teacher rating.

## Conclusion and discussion

The aim of the present study was to develop a better understanding of the interrelations between cognitive EF measures and behavioral EF ratings from both parents and teachers and to investigate their shared and unique influence on math and spelling achievement in first and second graders. A novel aspect of this study is the inclusion of EF ratings from multiple informants concurrently with cognitive EF performance measures to explain differences in school achievement. Furthermore, little research on the relation between EF and spelling has been published, especially in typically developing children using multiple modes of EF assessments. Analyses included IQ, a confounding factor for both school achievement and EF.

The main findings of this study were that the cognitive WM measure was correlated with its parent- and teacher-reported behavioral WM counterpart, and that all WM measures were significantly associated with school achievement. Furthermore, both the cognitive shifting and the teacher-reported behavioral shifting measure were also related to school achievement. None of the inhibition measures were significantly correlated with school outcome. Moderate correspondence was observed between parent's and teacher's ratings of children's behavioral EF. Cognitive performance and teacher's ratings of WM and shifting concurrently explained differences in spelling achievement. However, teacher's behavioral EF ratings did not explain any additional variance in math outcome above IQ and cognitive EF performance. Parent's behavioral EF ratings did not add any unique information to either outcome measure.

In comparing similar cognitive and behavioral aspects of EF, a significant and modest monotrait-multimethod correlation was only found between cognitive and behavioral ratings of WM. Thus, visual spatial working memory performance was somewhat linked to real-life WM problems that were observed by others, like forgetting what one was doing and having trouble remembering things at school or at home. Furthermore, modest correlations between parent and teacher ratings across all comparable EFs were found. These modest relations were consistent with findings by Toplak et al. (2013) and cross-informant findings in the related field of child psychopathology (Achenbach et al., 1987). Teachers perceived on average similar amounts of EF problem behavior compared to parents, but they only modestly agreed on which children had relatively more or less EF problems. This was also true for reporting the presence of a clinical level of EF problems (T-score ≥ 65). Teachers in our sample were, compared to a norm sample of peers, more likely to report a clinical level of EF problems than parents did; this was especially true for WM. The observed absent or modest monotrait-multimethod correlations suggest that each type of EF measure taps different aspects of EF across different situations and under variable conditions. Furthermore, the similar or even higher multitrait-monomethod correlations point to method variance caused by rater biases, e.g., halo and leniency bias, and test impurity problems.

### Math achievement

Based on the presented evidence we expected cognitive measures of WM, inhibition and shifting to be correlated to math achievement (e.g., Yeniad et al., 2013; Friso-van den Bos et al., 2013; Gerst et al., 2015; Ten Eycke and Dewey, 2016). Our study confirmed these findings, except for inhibition. Our finding that inhibition did not have a direct relation with math was in contrast to findings from a meta-analysis of 4–12-year-old children (Friso-van den Bos et al., 2013), and from recent studies in 9- to 11-year-olds (Gerst et al., 2015), and in 5–18 year-olds (Ten Eycke and Dewey, 2016), although the meta-analysis of Friso-van den Bos et al. (2013) also showed that WM had the strongest relation to math, and that inhibition and shifting showed the weakest relation. Furthermore, our findings also differed from previous findings linking inhibition to emerging math skills in preschoolers and kindergartners (e.g., Espy et al., 2004; Blair and Razza, 2007; Allan et al., 2014). Perhaps, only more extreme levels of inhibitory problems affect math outcome negatively, or inhibition is more likely to play a role in children with mathematical disorders or from economically disadvantaged families, which were included in the meta-analyses of Allan et al. (2014) and Friso-van den Bos et al. (2013). In fact, the meta-analysis of Friso-van den Bos et al. (2013) showed that children with math, psychological or physical problems have stronger associations between EF and math outcome. The children in our study were not at risk for mathematical problems nor inhibition problems, and predominantly came from families with medium to high socio-economic backgrounds. This study also showed that the influence of EF on math is in addition to the effect of IQ, which is in line with previous research (e.g., George and Greenfield, 2005; Alloway and Alloway, 2010; Preßler et al., 2013; Yeniad et al., 2013; Gerst et al., 2015; Dekker et al., 2016), and underscores the suggestion that IQ cannot be considered a proxy of EF or vice versa.

Based on the study of Gerst et al. (2015), we expected that only for WM a behavioral measure, most likely the teacher's rating, would add unique variance to the cognitive WM measure and IQ in explaining math performance. Unlike Gerst et al. (2015), we did not observe a similar impact for the teacher WM rating, nor for the parent rating of WM, although the latter measure was borderline significant. Nevertheless, comparable to Gerst et al. (2015), our results showed that none of the behavioral measures of inhibition or shifting added any unique variance explaining math outcome besides IQ. Thus, for math achievement we were able to confirm most of Gerst et al. (2015) findings in a younger age group, while also including parent EF ratings.

### Spelling achievement

Based on research about the relation between EF and spelling, we expected the cognitive measure of WM to be related to spelling outcome (e.g., Fischbach et al., 2013; Preßler et al., 2013; Becker et al., 2014). We could confirm that WM was related to spelling performance. Our results also extend the previous finding by Altemeier et al. (2008) that in typically developing first to fourth graders shifting ability is related to spelling, although we could not confirm their finding of a significant relation between inhibition and spelling. Inhibition and emerging writing skills have also been linked in preschoolers (Blair and Razza, 2007; McClelland et al., 2007; Brock et al., 2009). Altemeier et al. (2008) used a verbal word-color naming task to assess inhibition and shifting, while in our study we used nonverbal tasks. Perhaps, measures of verbal inhibition have a stronger association with spelling skills than non-verbal measures. Research in math, for example, has shown that visual spatial WM is more strongly related to learning something new, while verbal working memory is more related to learned math skills, which are typically evaluated through standardized achievement tests that are also used in this study (Van de Weijer-Bergsma et al., 2015). Similar differences across different stages of spelling attainment might also be observed for inhibition. Future research is needed to address the relative impact of verbal vs. visual spatial performance based EF measures in relation to various school outcomes and taking into account different stages of the learning process (e.g., acquiring or mastering).

No previous publications have considered the joint impact of different EF measures on spelling. We based our expectations, i.e., teacher's EF ratings having the biggest influence, and the cognitive measure of WM also adding variance, on the findings by Gerst et al. (2015) on another language related outcome, i.e., reading comprehension. In our study we found that both teacher behavioral ratings and cognitive measures of WM and shifting were related to spelling outcome, partially confirming our tentative hypotheses. Thus, real life application of WM and shifting skills at school helps to explain differences in spelling outcome concurrently with their cognitive counterparts. Spelling in this study was assessed through a dictation test, which might ask for different EF skills compared to a general math achievement test, although in first grade the math questions were also read out loud by the teacher. Perhaps attentional processes play a bigger role during dictation tests. Indeed, parent and teacher ratings of inattention in children with emotional and behavioral problems have previously been associated with behavioral EF ratings on the BRIEF (McAuley et al., 2010), which might partially explain the contribution of behavioral EF ratings concurrently with cognitive EF measures in explaining differences in spelling outcome.

In sum, although the ecological validity of cognitive performance-based tasks have been questioned, this study confirmed that cognitive EF measures actually explained most unique variance in math outcome compared to behavioral EF measures. This study also provides support for the ecological validity of performance- and teacher rating-based EF tasks by showing that both measures have a complementary role in identifying spelling achievement problems. Furthermore, both WM and shifting abilities were related to school achievement in general rather than to a specific domain.

Several study limitations need to be acknowledged. First of all, children from only two Dutch schools in the same provincial region were included in this study. One school from a rural area and a second school from a town that is part of the metropolis of the cities of Rotterdam and The Hague. Although the distributions of our independent and outcome measures seem to represent levels of typically developing children, with the exception of teacher reported level of clinical WM problems, it is clear that the children in our study are not representative as far as the educational level of their parents is concerned. Children from parents with a low educational level are underrepresented, and our results cannot be generalized to this group. Our low risk sample might have resulted in weaker relations between EF and school achievement than those found in other studies comprising at-risk samples (e.g., Waber et al., 2006; Gerst et al., 2015). Stronger associations between EF and math outcome exist in children with relatively more math, psychological or physical problems, as was shown in the meta-analysis of Friso-van den Bos et al. (2013). Secondly, the inclusion of more classes from more schools would have given more reliable estimates of random variation around the intercept for class. Thirdly, this study used a cross-sectional design, so we could not study the differential predictive power of the various EF measures nor the development of EF in relation to school achievement over time, which precludes any causal inferences. Finally, it might be possible that the inclusion of teacher-based math and spelling grades could have resulted in a different pattern of the relative contribution of each type of EF measure, as grades might share more variance with behavioral measures.

Despite these limitations, the observation that WM and shifting were related to spelling and math outcome, regardless of the child's IQ level, points in the direction of possible benefits from stimulating EF skills in young children in addition to extra domain specific instruction, to optimize school performance. There is some evidence that school-based and computerized interventions aimed at improving EF skills have promising cognitive outcomes in young children (Thorell et al., 2009; Diamond and Lee, 2011; Diamond, 2012; Wass, 2015), although questions remain concerning the actual causal mechanisms involved in improving school achievement. For example: To what extent do these interventions directly train academic achievement? Or to what level do these interventions improve EF by reducing EF suppressors like anxiety, depressive feelings, sleep deprivation or low physical activity level? (Jacob and Parkinson, 2015; Diamond and Ling, 2016). Other remaining questions are the transfer of EF skills, the heterogeneity or homogeneity of the training regime, how long benefits last, and which children benefit the most. There is some indication that younger children and children from at risk groups (e.g., economically disadvantaged background, poor EF) benefit more from EF training (Diamond, 2012; Wass, 2015). Nevertheless, identifying and monitoring each child's EF strengths and weaknesses, especially in the WM and shifting domain might help teachers and other caregivers to broaden their range of remedial intervention options to optimize school achievement. This study's findings also show that both types of EF measures, cognitive performance tasks and teacher's behavioral rating scales, complement each other in explaining spelling achievement and suggest that both could be used to identify likely candidates for additional support.

Future research is needed to cross-validate our final models, and to compare the impact of each type of EF measure across a wider age range of students, preferably longitudinally, to detect developmental differences, and across more school achievement domains, using both verbal and non-verbal cognitive EF measures. Also, within certain domains, e.g., mathematics, it might be informative to study independent aspects of math (e.g., factual, procedural, conceptual; Raghubar et al., 2010).

## Ethics statement

This study was carried out in accordance with the recommendations of the standards of the Ethical Committee of the Leiden Institute of Education and Child Studies with written informed consent from the parents of all subjects (minors). All parents of subjects (minors), gave written informed consent in accordance with the Declaration of Helsinki. The protocol was approved by the Ethics Committee of the Leiden Institute of Education and Child Studies at Leiden University (ECPW-2010/016).

## Author contributions

MD was involved in the conception and design of the work, data collection, data analysis and interpretation, drafting the article, critical revision of the article and gave her final approval of the version to be published. TZ was involved in data interpretation, critical revision of the article and gave his final approval of the version to be published. AS was involved in the design of the work, data collection, data interpretation, critical revision of the article and gave her final approval of the version to be published. HS was initiator of the Curious Minds study and was involved as project leader in the conception and design of this work, data interpretation, critical revision of the article and gave her final approval of the version to be published.

## Funding

This research is funded by the Curious Minds Program, which is supported by the Dutch Ministry of Education, Culture and Science and the National Platform Science & Technology.

### Conflict of interest statement

The authors declare that the research was conducted in the absence of any commercial or financial relationships that could be construed as a potential conflict of interest.
